# Supplementary material for: Transcriptomic Insights into Mechanisms of Early Seed Maturation in the Garden Pea (Pisum sativum L.)
Source: Cells. 2020 Mar 23;9(3):779. doi: 10.3390/cells9030779 (PMC7140803; doi:10.3390/cells9030779)

Correlation Spanning Tree on module data (dmap)

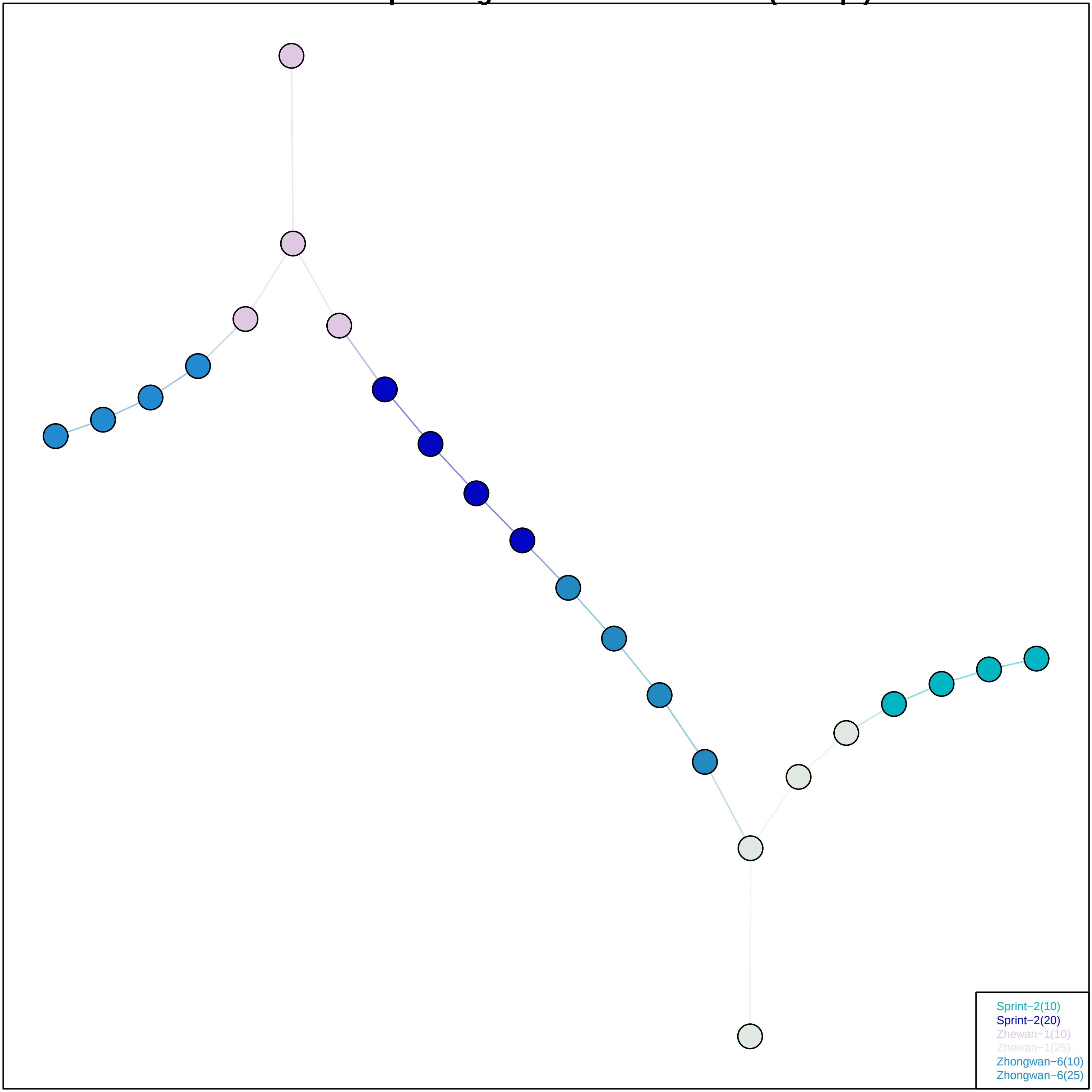

Correlation Spanning Tree & silhouette scores on module data (dmap)

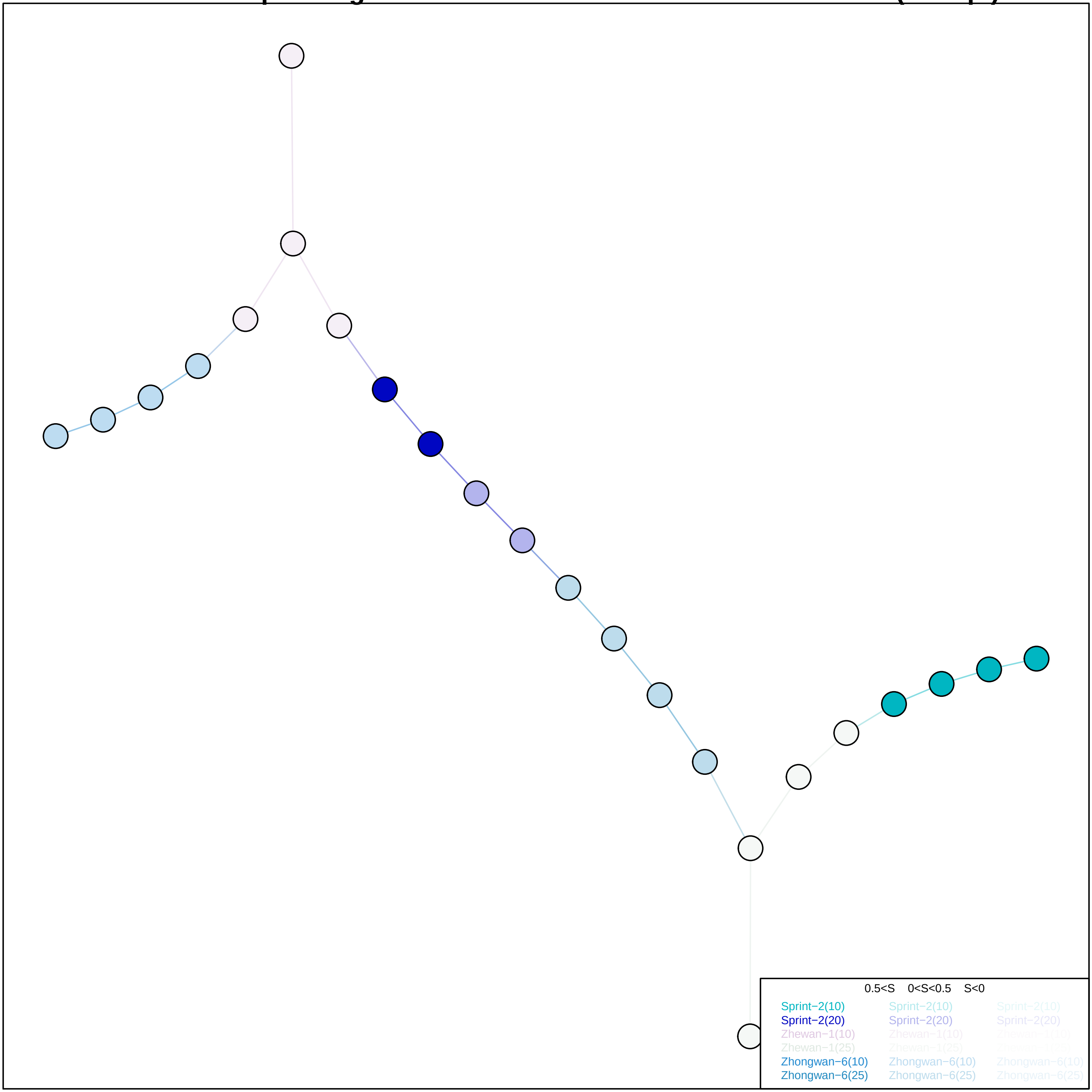

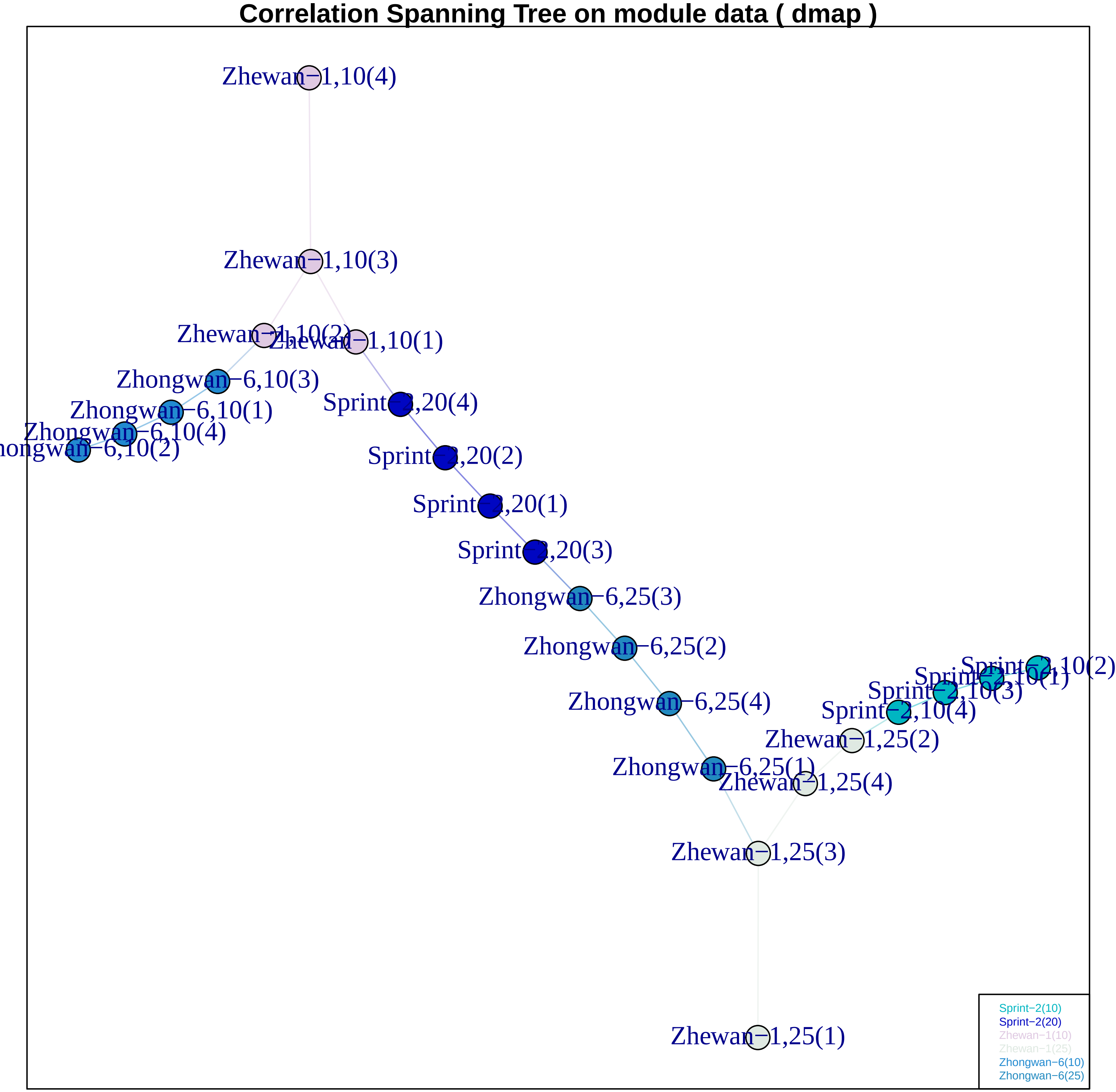
Correlation Spanning Tree on module data (dmap)
Zhewan−1,10(4)
Zhewan−1,10(3)
Zhewan−1,10(2)
Zhewan−1,10(1)
Zhongwan−6,10(3)
Zhongwan−6,10(1)
Zhongwan−6,10(4)
hongwan−6,10(2)
Sprint−2,20(4)
Sprint−2,20(2)
Sprint−2,20(1)
Sprint−2,20(3)
Zhongwan−6,25(3)
Zhongwan−6,25(2)
Zhongwan−6,25(4)
Zhongwan−6,25(1)
Sprint−2,10(2)
Sprint−2,10(1)
Sprint−2,10(3)
Sprint−2,10(4)
Zhewan−1,25(2)
Zhewan−1,25(4)
Zhewan−1,25(3)
Zhewan−1,25(1)
Sprint−2(10)
Sprint−2(20)
Zhewan−1(10)
Zhewan−1(25)
Zhongwan−6(10)
Zhongwan−6(25)

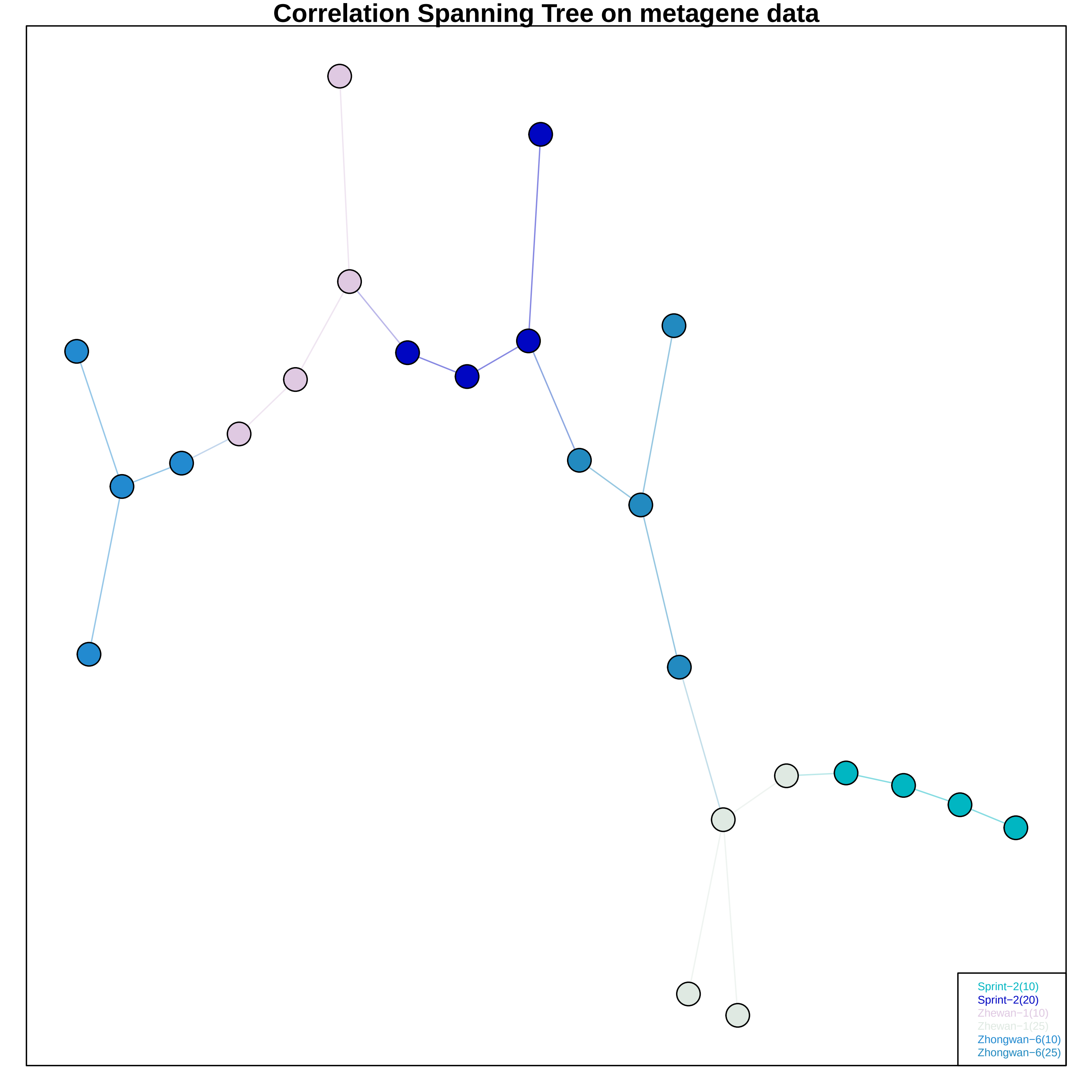

Correlation Spanning Tree on metagene data
Sprint−2(10)
Sprint−2(20)
Zhewan−1(10)
Zhewan−1(25)
Zhongwan−6(10)
Zhongwan−6(25)

Correlation Spanning Tree & silhouette scores on metagene data

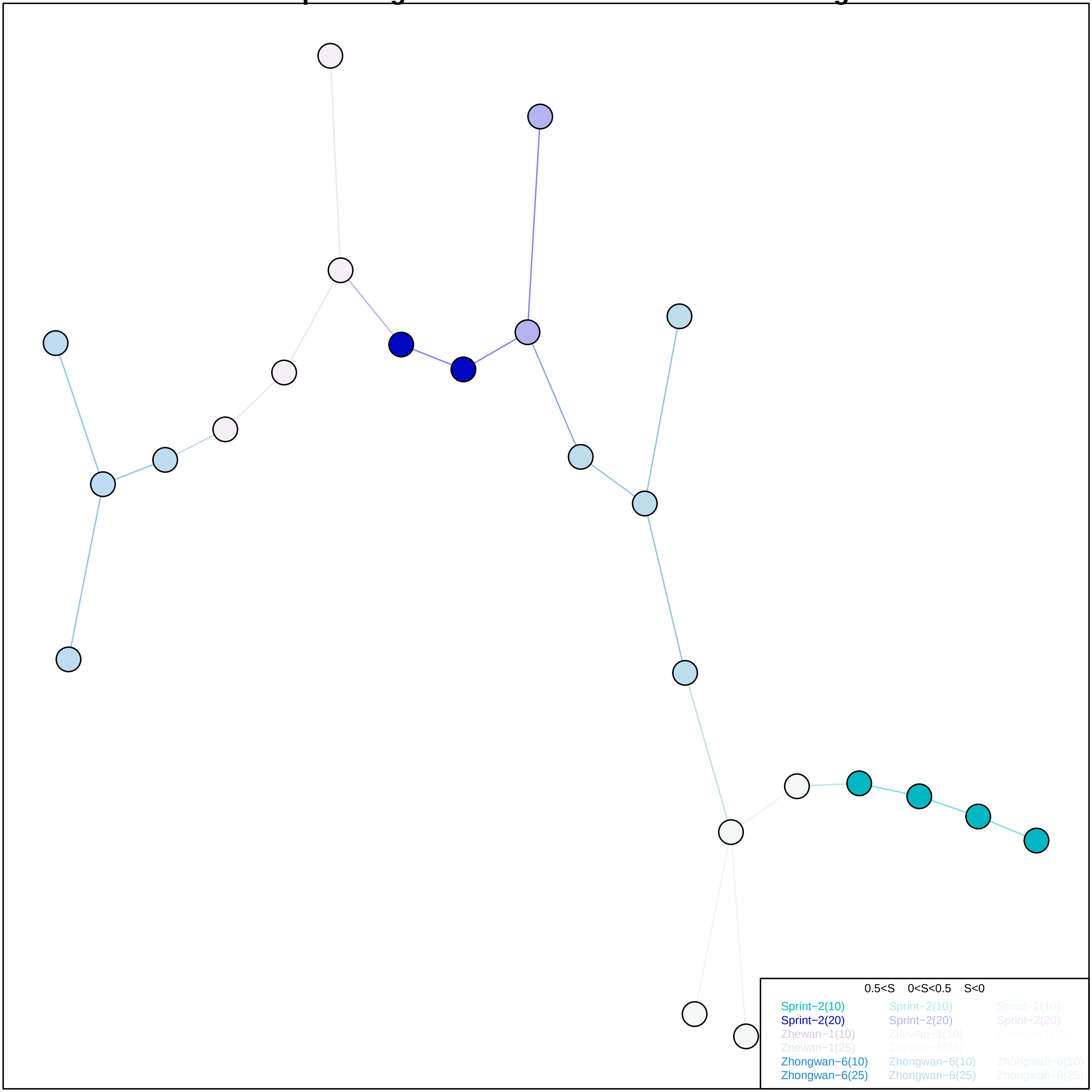

Correlation Spanning Tree on metagene data

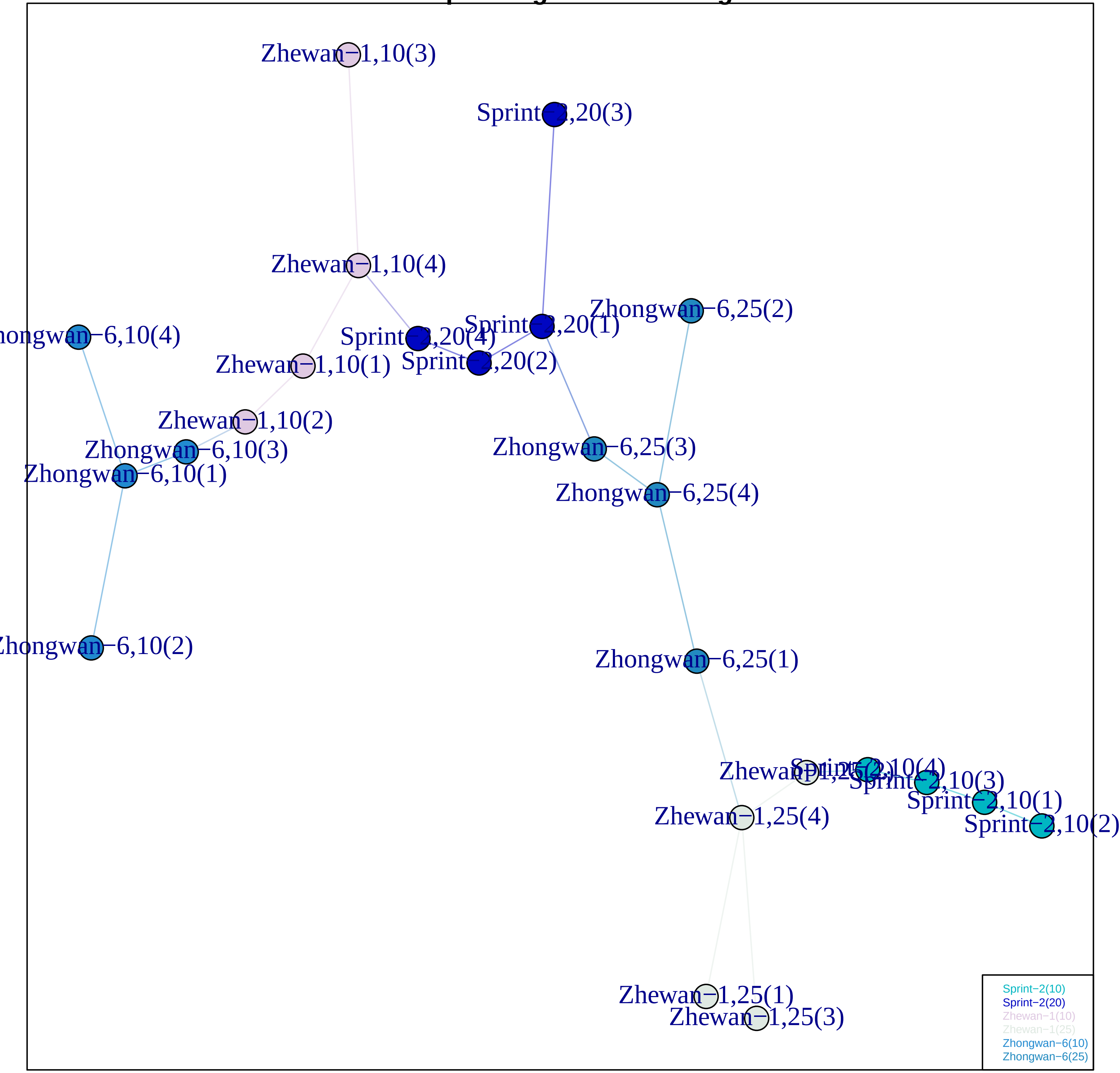

Supplement: Supplementary file 1 [file cells-09-00779-s001.zip › Supplementary materials/FileS4.zip!/FileS3/TFs - Results/Sample Similarity Analysis/Correlation Spanning Tree.pdf]
